# Terpenes and Phenylpropanoids with Potent Toxicity on Two Tephritid Fruit Flies and Reduced Impact to a Non-Target Parasitoid

**DOI:** 10.3390/insects17080774

**Published:** 2026-07-27

**Authors:** Letícia Jansen Medeiros, Michele Trombin de Souza, Mireli Trombin de Souza, Christian Peter Demari, Gabriel Radtke Abib, Leandro do Padro Ribeiro, Vanessa Nogueira Soares, Dori Edson Nava, Daniel Bernardi

**Affiliations:** 1 Department of Plant Health, Federal University of Pelotas, Pelotas 96010-610, Rio Grande do Sul, Brazil; leticia.jansen.m@gmail.com (L.J.M.); mirelitrombin@gmail.com (M.T.d.S.); christiandemari@hotmail.com (C.P.D.); gabriel.abib.bio@gmail.com (G.R.A.); vnsoares@gmail.com (V.N.S.); 2 Research Center for Family Agriculture, Agricultural Research and Rural Extension Company of Santa Catarina, Chapecó 89801-970, Santa Catarina, Brazil; micheletrombin@gmail.com (M.T.d.S.); leandroribeiro@epagri.sc.gov.br (L.d.P.R.); 3 Embrapa Temperate Climate, Pelotas 96010-971, Rio Grande do Sul, Brazil; dori.edson-nava@embrapa.br

**Keywords:** phytoconstituents, lethal, sublethal toxicities, selectivity, sustainable agriculture

## Abstract

Tephritid flies, highlighting *Anastrepha fraterculus* and *Ceratitis capitata*, cause substantial economic losses and trigger strict quarantine measures worldwide. In response, research is looking for eco-friendly alternatives and, in this study, we evaluated 7 terpenes/terpenoids (α-pinene, β-pinene, β-caryophyllene, γ-terpinene, ρ-cymene, citronellal and geropine) and 3 phenylpropanoids (isoeugenol, methyl cinnamate and coumarin) commonly found in a diverse group of aromatic plants tested against *A. fraterculus* and *C. capitata* adults. Subsequently, their selectivity for the egg–larvae endoparasitoid *Doryctobracon areolatus* was also assessed. Ingestion and topical application bioassays (range = 100–6000 mg L^−1^) revealed that isoeugenol caused the highest mortality (≥75%) in both fruit fly species. Concentration–response indicated a similar toxicity of this compound to *A. fraterculus* (ingestion: LC_50_ ≅ 34.2 mg L^−1^, LC_90_ ≅ 105.7 mg L^−1^; topical: LC_50_ ≅ 33.7 mg L^−1^, LC_90_ ≅ 121.3 mg L^−1^) and *C. capitata* (ingestion: LC_50_ ≅ 33.2 mg L^−1^, LC_90_ ≅ 125.5 mg L^−1^; topical: LC_50_ ≅ 31.4 mg L^−1^, LC_90_ ≅ 129.2 mg L^−1^). None of the tested compounds (α-pinene, β-pinene, ρ-cymene, β-caryophyllene, isoeugenol, γ-terpinene, coumarin, citronellal, geropine and methyl cinnamate) reduced the number of oviposition punctures on grape berries by either fruit fly species. In contrast, all compounds caused lower mortality in *D. aerolatus* (≤17%) and did not impair parasitism, with a range of 43.21–62.13% among the tested compounds. Overall, our findings indicate that selected phytocompounds, particularly isoeugenol and citronellal, show strong insecticidal activity against *A. fraterculus* and *C. capitata* while having low toxicity to *D. areolatus*, supporting their use into sustainable pest management programs.

## Simple Summary

Fruit flies are serious pests of fruit crops because females lay eggs inside fruits, where the developing larvae cause damage, reduce market value, and create barriers to international trade. This study evaluated ten plant-derived compounds as possible alternatives to insecticides for controlling two important fruit fly species, *Anastrepha fraterculus* and *Ceratitis capitata*. The compounds were tested through ingestion and direct contact, and their effects on egg-laying were also assessed. In addition, their safety to *Doryctobracon areolatus*, a beneficial wasp that naturally attacks fruit fly species, was investigated. Isoeugenol and citronellal caused the highest mortality in both fruit fly species, with effects comparable to those of the commercial insecticide used as a reference. However, the tested plant compounds did not reduce the number of egg-laying punctures on grapes. Most importantly, they caused low mortality in the beneficial wasp and did not reduce its ability to parasitize fruit fly larvae. These findings indicate that selected plant-derived compounds, especially isoeugenol and citronellal, may support safer and more sustainable fruit fly management while preserving an important biological control agent.

## 1. Introduction

Fruit flies (Diptera: Tephritidae) pose a phytosanitary threat to the global trade in fruits and vegetables production. Their direct damage is caused by oviposition punctures and larval feeding inside the fruit, which promote tissue breakdown, premature fruit drop, secondary microbial infections, and loss of commercial value [1,2,3]. As a result, infested products become unfit for consumption and are subject to quarantine restrictions imposed by importing countries. Among the globally relevant species, *Anastrepha fraterculus* (Wiedemann, 1830) is restricted to the American continent, from northern Mexico to southern Argentina [4], while *Ceratitis capitata* (Wiedemann, 1824) has a cosmopolitan distribution, being native to Africa and currently widespread in tropical and subtropical regions of all continents [5]. Both species are present in Brazil and exhibit ecological niche overlap, a wide range of hosts, high biological plasticity, and, consequently, difficulty in control [6]. Their economic impacts exceed two billion dollars globally; in Brazil, fruit flies also cause substantial losses, including approximately 5% of apple production, equivalent to about 60,000 tons and US$110 million, attributed to *A. fraterculus* in southern orchards, as well as annual losses estimated at US$242 million associated with *C. capitata* [7]. As a result, many fruit-producing and importing countries impose strict quarantine restrictions, requiring post-harvest treatments such as fumigation, heat treatment, and irradiation [8]. In response, Integrated Tephritidae Management Programs include strategies for the eradication or suppression of the species, using synthetic insecticides [9,10], as well as Sterile Insect Technique and Male Sterilization [8], and the use of biological control via parasitoids, predators [4,11], and entomopathogenic microorganisms [11]. Recently, research has sought alternative and environmentally friendly tools for adoption—in particular, terpenic and phenylpropanoid phytochemicals have emerged as a promising alternative [8]. This is because, in general, they exhibit low toxicity to natural enemies, degrade rapidly in the environment, have a reduced environmental impact [8,12], and are compatible with both conventional and organic agriculture [13].

From a biochemical perspective, terpenes are derived from isoprene units (C_5_H_8_) and include monoterpenes, sesquiterpenes, and diterpenes synthesized via the mevalonate or methyl erythritol phosphate pathways [14], while phenylpropanoids are aromatic compounds derived from the chiquimic acid pathway [15]. These phytoconstituents may also contain a variety of functional groups, such as oxygenated compounds (monoterpene and sesquiterpene alcohols, aldehydes, ketones, and esters), sulfur and nitrogen-containing compounds (thioesters, sulfides, isothiocyanates, and nitriles), hydrocarbons, and others [14,15]. They encompass multifunctional chemical constituents demonstrating potent acaricidal [16], insecticidal, anti-feeding [17,18], growth-regulating, behavior-modulating activity [19,20], and repellent effects [21,22,23,24]. However, although many studies show promising results for the use of terpenes and phenylpropanoids in the control of various taxonomic groups, including *A. fraterculus* and *C. capitata* [8,20,25,26], the number of commercial products based on structurally similar phytoconstituents remains extremely low [19,20,25,26]. In addition, challenges to the commercial use of these products include certain legislative barriers [21,26,27], since extensive toxicological studies are typically required prior to the approval of a botanical derivative, making commercial registration unfeasible in some cases [17,27,28]. Furthermore, some of these compounds are sensitive to oxygen, light, and high temperatures [16,18], requiring the adoption of formulation processes.

Although the underlying mechanisms of toxicity of terpenes and phenylpropanoids have been elucidated [29], understanding their toxicity to both insect pests and non-target organisms is extremely important to ensure the compatibility of new products to be launched on the market with biocontrol agents [30,31]. Among the biocontrol measures for tephritids, *Doryctobracon areolatus* (Szépligeti, 1911) (Hymenoptera: Braconidae) is a solitary endoparasitoid of eggs and early-stage larvae. Native to South America, it is currently distributed throughout all Brazilian fruit-growing regions [1]. Recently, the Brazilian Ministry of Agriculture and Livestock (MAPA) registered *D. areolatus* as a biological control agent for fruit flies [4,32]. However, *D. areolatus* is highly sensitive to synthetic insecticides used in the management of *A. fraterculus* and *C. capitata* [32], a fact that highlights the need to diversify integrated tephritids management strategies and mitigate some of the negative impacts of synthetic insecticides on the agroecosystem [4,8].

Given this context, we hypothesized that preselected terpenoid and phenylpropanoid phytochemicals would exhibit insecticidal activity and/or oviposition deterrent effects against *A. fraterculus* and *C. capitata*. As a secondary hypothesis, we predicted a lesser impact on *D. areolatus*, thereby promoting the ecological balance of agroecosystems. To test these hypotheses, we evaluated the lethal toxicity, via ingestion and topical application, of 10 phytochemicals (α-pinene, β-pinene, β-caryophyllene, γ-terpinene, ρ-cymene, citronellal, geropine, isoeugenol, methyl cinnamate and coumarin) on adults of *A. fraterculus* and *C. capitata*. Next, we analyzed the selectivity of these compounds against *D. areolatus* through toxicity bioassays (ingestion and topical application) and evaluated the effects of the treatments on larval endoparasitism in tephritids. The results of this study aim to contribute to the development of sustainable tephritids management programs, offering alternatives to overcome resistance to synthetic insecticides and the possibility of generating bio-inputs to be made available to the market.

## 2. Materials and Methods

### 2.1. Tested Terpenes and Phenylpropanoids

Terpenes and phenylpropanoids, including hydrocarbon monoterpenes (α-pinene, β-pinene, γ-terpinene, and ρ-cymene), an oxygenated monoterpene (citronellal), sesquiterpene hydrocarbons (β-caryophyllene), phenylpropanoids (isoeugenol and coumarin), and aromatic esters (geropine and methyl cinnamate) were supplied by Geroma do Brasil Indústria e Comércio Ltda. (Ponta Grossa, Paraná, Brazil). All isolates had a purity of ≥95%, according to the suppliers’ analytical specifications. The chemical structures of these compounds were also confirmed by injection of another commercial standard solution from Sigma-Aldrich S/A (St. Louis, MO, USA), following protocols suggested by Souza et al. [33].

### 2.2. Bioassays

All bioassays were conducted under controlled conditions (temperature of 25 ± 2 °C, 70 ± 10% RH, and a 12 h photoperiod) using a completely randomized design. Regardless of the route of exposure to which the insects were subjected, the treatments consisted of pure isolates at different concentrations (range = 100–6000 mg L^−1^) diluted in acetone, as well as the spinosyn-based positive control (Spinetoram—7.5 mg a.i. L^−1^; Delegate™ 250 WG, Dow AgroSciences, Santo Amaro, São Paulo, Brazil) dispersed in distilled water. The distilled water used to solubilize the treatments was considered the negative control.

#### 2.2.1. Bioassays

Specimens of *A. fraterculus* and *C. capitata* used in the bioassays were obtained from colonies established at the Entomology Laboratory of Embrapa Clima Temperado. Adult flies were housed in acrylic cages (57 cm long × 39 cm wide × 37 cm high) and provided *ad libitum* with distilled water and an artificial diet composed of wheat germ, hydrolyzed yeast, and sugar (1:1:3, *v*/*v*/*v*). Eggs were obtained by placing a voile-type fabric on one side of the adult cages, and females were attracted to the oviposition substrate by fluorescent lamps (360 nm and 40 watts). Twenty-four h after oviposition, the eggs were placed on an artificial diet, and larval development was allowed to proceed until the end of the third instar, at which point the larvae were rinsed under running water and placed for pupation in moistened vermiculite in trays (25 cm long × 7.5 cm high), where they were kept until emergence.

In turn, *D. areolatus* were reared using early-stage larvae of *A. fraterculus* or *C. capitata* as hosts. For this purpose, the tephritids larvae were exposed to female parasitoids in rearing cages for 24 h; thereafter, the parasitized larvae were transferred to an artificial diet according to the protocol described above until proper pupation. The pupae were then collected and placed in containers with moistened vermiculite, where they remained until the parasitoids emerged. Adults of *D. areolatus* were fed *ad libitum* with a 20% (*v*/*v*) aqueous honey solution.

#### 2.2.2. Lethal Toxicity to *A. fraterculus* and *C. capitata*

All bioassays (ingestion or topical application) were conducted under controlled conditions, as described previously. In the ingestion bioassay, 10 pairs of 8-day-old *A. fraterculus* and *C. capitata* were divided into groups (sample units) and released into cages made of transparent plastic cups (1 L), inverted onto a plastic Petri dish (25 cm in diameter) and sealed at the top (bottom of the cup) with a voile mesh to allow gas exchange and prevent the insects from escaping, as illustrated in Souza et al. [34]. After the treatments were diluted, they were provided to the tephritids inside the cages via capillary action on hydrophilic cotton rolls in glass vials (10 mL). After 24 h, the treatments were removed, and the adults were fed an artificial diet and distilled water until the end of the evaluation period. For each treatment, 10 replicates (cages) were used, each consisting of 10 adults (*n* = 100) of each species. Mortality was assessed at 24 h intervals up to 120 h after the start of treatment. The insects were considered dead when no movement occurred after being touched with a fine brush.

In the topical application bioassay, adults of *A. fraterculus* and *C. capitata* were divided into groups of 10 adults of each species (10 days old) and placed in transparent glass tubes (2.5 cm in diameter × 8 cm in length) sealed with cotton plugs. Subsequently, the insects were anesthetized in the freezer for 30 s and placed on a glass Petri dish (9 cm in diameter) lined with filter paper. Subsequently, the insects were sprayed using a Vonder^®^ airbrush (Curitiba, Brazil), calibrated to a pressure of 30 psi, with a 0.3 mm nozzle, which allowed for the deposition of 1.5 mg cm^−2^ of each solution. In these bioassays, 1 mL of the solution was applied to the insects. Afterward, the tephritids were housed in cages made from transparent plastic cups (1 L) sealed with a ventilation lid made of voile-type mesh, as described previously. The adults were fed an artificial diet and distilled water in the same manner described previously, until the end of the evaluation period. Mortality was assessed as described previously, and for each treatment, 10 replicates were used with 10 adults per replicate (*n* = 100) of each species.

#### 2.2.3. Concentration–Response Curves

All treatments were subjected to a new bioassay to estimate the lethal concentration required to kill 50% and 90% of the exposed tephritids (LC_50_ and LC_90_, respectively). To this end, eight concentrations of each treatment were tested, ranging from 100 to 6000 mg L^−1^. The exposure and evaluation procedures, as well as the mortality criteria, were the same as those used in the lethal toxicity tests (ingestion and topical application bioassays). In both bioassays, 10 replicates were conducted with 10 adults of each species (*n* = 100) for each concentration of each treatment evaluated.

#### 2.2.4. Ovideterrent Effect of the Tested Compounds on *A. fraterculus* and *C. capitata*

To evaluate the effects of the tested compounds and their respective controls (positive and negative) on the oviposition behavior of *A. fraterculus* and *C. capitata*, adults of both species were subjected to forced-choice bioassays, using ripe grapes of the ‘Italia’ variety (intact and free of insecticide contamination) as an oviposition substrate. To this end, the grape berries were immersed in insecticide solutions diluted in water for 5 s and subsequently dried on filter paper for 3 h. After this time, the grape berries were placed inside a semi-transparent cage made of transparent plastic cups (1 mL), inverted over a Petri dish (8 cm in diameter) (one per cage). Subsequently, two pairs of 13-day-old *A. fraterculus* and *C. capitata* were placed inside. The insects were fed an artificial diet + distilled water.

After 24 h, the adults were removed, and the grape berries were placed individually inside plastic cups (50 mL) on a layer of vermiculite (1 cm), sealed at the top with Parafilm™, and stored in a climate-controlled room. After five days, the number of “punctures”—that is, oviposition marks caused by females of *A. fraterculus* and *C. capitata*—was counted. The number of punctures was counted using a stereoscopic microscope (40×). The experimental design was completely randomized with 50 berries per treatment.

#### 2.2.5. Lethal and Sublethal Toxicity on the Parasitoid *D. areolatus*

To assess the lethal effect of the treatments on the parasitoid *D. areolatus*, the LC_90_ values determined from the concentration–response curves after 120 h of exposure to each treatment in toxicity assays on adults of *A. fraterculus* were used. To this end, adults of *D. areolatus* were subjected to both exposure procedures (ingestion and topical application), as described previously. Insect mortality was assessed after 120 h of exposure using the same criteria used to assess mortality in adult flies.

To assess the sublethal effects of the treatments, the parasitoids that survived after 120 h were used in the bioassays (one pair per cage). For this purpose, third-instar larvae of *C. capitata* were offered for 10 consecutive days (10 larvae per female in a mixture of cornmeal and wheat fiber). After 7 h of exposure, the larvae were removed and transferred to a wheat germ-based diet to complete larval development. After 4 days, the larvae were transferred to plastic containers (80 mL) containing cornmeal (1 cm) as a substrate for pupation. After approximately 4 days, the pupae were separated from the substrate until adult emergence in a climate-controlled room. When the insects (*C. capitata* or *D. areolatus*) emerged, they were counted. The pupae that remained intact were dissected to check for the presence of unmerged flies or parasitoids to determine the parasitism rate. For each treatment, parasitism (%) was determined by dividing the total number of “parasitoid” offspring by the total number of pupae offered, multiplied by 100.

### 2.3. Statistical Analysis

Generalized linear models (GLMs) from the exponential family of distributions were used to analyze the variables under study [35]. The quality of the fit was assessed using semi-regular probability plots with simulation envelopes [36]. When significant differences were detected between treatments, multiple comparisons (Tukey’s post hoc test, *p* < 0.05) were performed using the glht function of the Multicomp package, with adjustment of *p*-values. All analyses were performed using the statistical software “R”, version 9.2 [37].

A binomial model with a complementary log-log link function (Gompit model) was fitted to estimate the lethal concentrations (LC_50_ and LC_90_), using the Probit procedure in SAS software, version 9.2 [38,39].

## 3. Results

### 3.1. Lethal Toxicity

**Figure 1:**
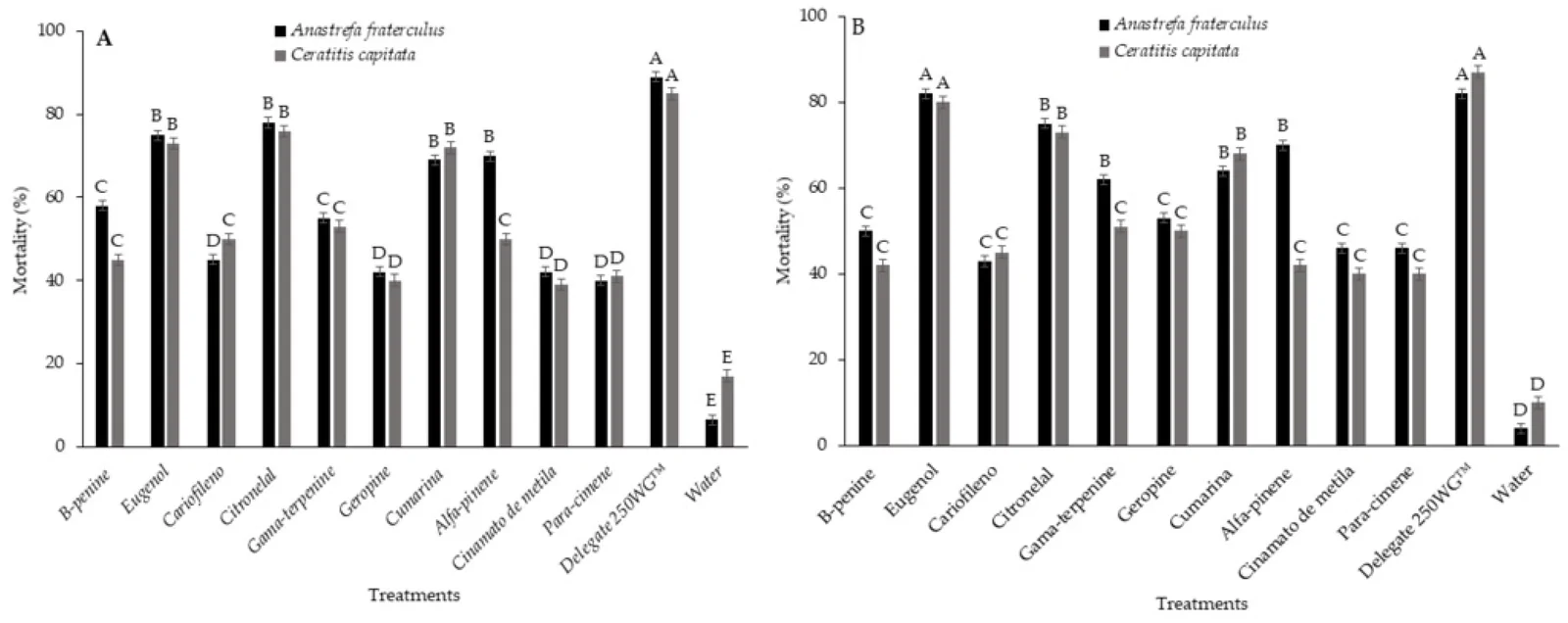
Mortality rate (±SE) of *Anastrepha fraterculus* and *Ceratitis capitata* exposed for 120 h to terpenes and phenylpropanoids via ingestion (**A**) and topical application (**B**). Means followed by different letters in each figure indicate significant differences between treatments (one-way ANOVA, Tukey test, *p* < 0.05).

The mortality of adults of *A. fraterculus* (F = 12.11, df = 11, 109, *p* > 0.0001) and *C. capitata* (F = 8.13, df = 11, 109, *p* > 0.0001) differed significantly depending on the compounds tested and the mode of exposure for each pest species. In the ingestion bioassay (Figure 1A), the highest mortality rates were observed with isoeugenol and citronellal, which were generally equitoxic to Delegate 250 WG™ (positive control), exceeding 70% mortality for both pest species under the different exposure conditions. Intermediate mortality rates for *A. fraterculus* and *C. capitata* were recorded for β-pinene, γ-terpinene, coumarin, and α-pinene, while geropine, methyl cinnamate, and ρ-cymene resulted in reduced mortality, but still statistically different from the negative control (distilled water) (Figure 1A).

In topical application (Figure 1B), a similar pattern was observed for *A. fraterculus* and *C. capitata* with isoeugenol, causing mortality in both species (≥80%) comparable to that of the positive control (Delegate 250 WG™). Moderate mortality was observed for citronellal and coumarin, while β-pinene, β-caryophyllene, geropine, methyl cinnamate, and ρ-cymene showed lower efficacy for both species of Tephritidae (Figure 1B).

### 3.2. Lethal Concentration

Probit analyses revealed that all tested compounds were toxic to *A. fraterculus* and *C. capitata*, with acceptable fits for all models in the ingestion bioassay (Table 1). For *A. fraterculus*, LC_50_ values ranged from 17.11 to 42.19 mg L^−1^, and LC_90_ values ranged from 87.13 to 123.34 mg L^−1^. Among the treatments tested, β-pinene and Delegate 250 WG™ exhibited the lowest LC_50_ values, while the other compounds tested required higher concentrations to achieve comparable mortality via ingestion (Table 1).

Similarly, for *C. capitata*, LC_50_ values ranged from 21.14 to 39.10 mg L^−1^, and LC_90_ values ranged from 74.51 to 132.44 mg L^−1^. However, the concentration–response curves and the overlap of confidence intervals did not differ between treatments, except for the LC_90_ of Delegate 250 WG™ (74.51 mg L^−1^), which had a lower concentration via ingestion (Table 1). In the topical application bioassay (Table 2), LC_50_ values ranged from 21.44 to 37.18 mg L^−1^, and LC_90_ values ranged from 90.11 to 127.89 mg L^−1^ for *A. fraterculus*, while in *C. capitata* LC_50_ values ranged from 23.17 to 34.15 mg L^−1^, and LC_90_ values ranged from 87.11 to 144.23 mg L^−1^. However, for both tephritids, no statistical differences were observed between the treatments, differing only from Delegate 250 WG™ (LC_50_ = 21.44 mg L^−1^ and LC_90_ = 90.11 mg L^−1^ for *A. fraterculus*; LC_50_ = 23.17 mg L^−1^ and LC_90_ = 87.11 mg L^−1^ for *C. capitata*) which had the lowest concentrations via ingestion (Table 2).

### 3.3. Repellency of the Tested Fruit Preserves Against A. fraterculus and C. capitata

The number of punctures formed by the oviposition of *A. fraterculus* and *C. capitata* differed significantly among treatments [One-way ANOVA; (*A. fraterculus*: F = 22.45, df = 11, 330, *p* < 0.0001) and *C. capitata* (F = 17.56, df = 11, 330, *p* < 0.0001); Table 3)]. For *A. fraterculus*, all tested compounds resulted in a similar number of punctures, ranging from 1.30 to 1.55 punctures, with no significant differences relative to the negative control [distilled water (1.89 punctures); Table 3]. In contrast, the commercial insecticide Delegate™ completely inhibited oviposition by *A. fraterculus*, differing significantly from the other treatments.

For *C. capitata*, the number of punctures associated with the pure compounds and the negative control (distilled water) ranged from 0.75 to 1.10 punctures, with no significant differences among the treatments. As observed for *A. fraterculus*, Delegate™ 250 WG also completely suppressed oviposition by *C. capitata*, forming a group statistically distinct from the other treatments. Overall, in both species of tephritids, although the pure compounds did not significantly reduce the number of punctures, the synthetic insecticide Delegate™ 250 WG consistently prevented oviposition.

### 3.4. Selectivity Regarding Doryctobracon areolatus

Adult mortality of *D. aerolatus* differed significantly among treatments in both the ingestion bioassay and the topical application (one-way ANOVA; ingestion: F = 9.11; df = 11, 240, *p* < 0.0001; topical: F = 10.12; df = 11, 240; *p* < 0.0001). In the ingestion bioassay, mortality of *D. aerolatus* caused by the compounds ranged from 4.11% to 17.12%. Isoeugenol, β-caryophyllene, citronellal, coumarin, α-pinene, methyl cinnamate, and ρ-cymene induced higher mortality levels and were similar to one another, while pinene, terpinene, and geropine did not differ from the negative control (distilled water). On the other hand, the synthetic insecticide Delegate™ 250 WG caused significant mortality (38.10%) and formed a statistically distinct group.

In the topical application bioassay, mortality ranged from 10.44% to 14.24% for most of the tested compounds. Once again, Delegate™ 250 WG induced the highest mortality (31.90%), differing significantly from all other tested compounds (Table 4). However, when considering the parasitism rate of *D. aerolatus*, the values ranged from 43.21% to 62.13% among the pure isolates, with no differences observed between the treatments (Table 4).

## 4. Discussion

This study provides evidence of the lethal and sublethal effects of 10 phytochemicals commonly found in essential oils on populations of *A. fraterculus* and *C. capitata*, as well as their reduced impact on the parasitoid *D. aerolatus*. Terpenes and phenylpropanoids can cause mortality at different developmental stages and malformation in tephritids [40], oviposition deterrence [41,42], and attract females and males [43], with potential for use in attract-and-kill strategies. Among the compounds examined, the monoterpenes α-pinene and β-pinene were the phytochemicals reported in the literature as being ineffective against females, while males gain an advantage in mating [44,45], whereas ρ-cymene is reported as attractive to both sexes [43]. To the best of our knowledge, the other terpenes and phenylpropanoids analyzed had not yet been investigated against the targets used. When the compounds are considered individually, their behavioral relevance appears to depend strongly on chemical identity, insect species, concentration and whether they are tested alone or as part of a volatile blend. α-pinene and β-pinene are common monoterpene hydrocarbons found in several host plant volatile profiles. Although α-pinene has been associated with sexual stimulation and increased mating success in some tephritids, its isolated attractiveness to *A. fraterculus* and *C. capitata* remains poorly supported. β-caryophyllene, a sesquiterpene hydrocarbon, may contribute to host recognition in complex plant-odor mixtures; however, when tested alone, it did not stimulate male calling behavior in *A. fraterculus* [8]. Similarly, γ-terpinene is generally interpreted as a component of complex fruit volatile blends rather than as a strong isolated attractant for these species [8]. In contrast, ρ-cymene has been reported as an attractant for *C. capitata*, with previous studies showing attraction of both males and females to mango airborne terpenes containing this compound [8,43]. Citronellal, an oxygenated monoterpene, has been associated with toxic or repellent effects that may vary with dose and formulation [8]. Among the phenylpropanoid-related compounds, methyl cinnamate deserves attention because of its well-established role in tephritids’ chemical ecology, especially as a male attractant in methyl-eugenol-responsive species, supporting its potential use in lure-based and attract-and-kill strategies [8]. Finally, the behavioral role of geropine in tephritids remains unclear and requires further investigation. Although the 10 selected compounds differ in their functional groups, citronellal and isoeugenol induced high levels of mortality in *A. fraterculus* and *C. capitata*, similar to those observed for the commercial insecticide Delegate™ 250 WG, while monoterpene hydrocarbons, including α-pinene, β-pinene, γ-terpinene, and ρ-cymene, exhibited lower efficacy. This pronounced bioactivity of citronellal and isoeugenol is consistent with previous studies reporting greater insecticidal potential of oxygenated monoterpenes [45] and phenylpropanoids [43,46] against tephritids. In fact, the lipophilic and volatile nature of many essential-oil constituents may facilitate their penetration through the insect cuticle, contributing to rapid insecticidal effects and, in some cases, knockdown response [12,21,47]. Eugenol derivatives, such as methyl eugenol, are substances with bioactivity against *C. capitata* [48] and, in addition, also play a role in the production of male pheromones [48,49,50], acting as agonists of ionotropic γ-aminobutyric acid (GABA) receptors (GABARs) [51] and by altering the permeability of neural membranes [52]. In contrast, monoterpene hydrocarbons generally have lower toxicity due to their low molecular weight and high volatility when applied individually [45], although they can act synergistically with oxygenated functional groups in blends [31,53]. Thus, enhanced baits have been developed through a combination of multiple chemotactic compounds that act synergistically [54]. For example, α-pinene and β-pinene, both monoterpene hydrocarbons, lack polar functional groups, which may limit their interaction with targets in the nervous system and reduce their persistence on the insect cuticle [45].

In this study, we observed similar mortality rates between *A. fraterculus* and *C. capitata*, which may be interpreted as an indication of comparable susceptibility of these species to the tested phytochemicals, consistent with previous research on the responses of tephritids to terpenes and phenylpropanoids [44,45]. Additionally, the subtle interspecific differences detected for some treatments may reflect a species-specific relationship, which is potentially related to differences in cuticle composition, detoxification enzymes, or behavioral responses [55]. Unlike other Diptera [17,18], we observed higher mortality in ingestion bioassays compared to topical application. Likely, after ingestion, the bioactive compounds are rapidly absorbed by the intestinal epithelium, allowing for systemic distribution and prolonged interaction with physiological targets [56]. From a practical standpoint, ingestion toxicity is important for many “attract and kill” approaches targeting tephritids [8]. In contrast, topical application may underestimate toxicity due to the high volatility of phytoconstituents and limited penetration through the insect cuticle, as previously observed [21,47], an aspect that can be enhanced through formulation procedures [16,57].

In addition to lethal toxicity, behavioral aspects of *A. fraterculus* and *C. capitata* should also be considered, including the oviposition stimulation or deterrence [41,42,54] and attraction mediated by plant volatiles or sex-related chemical cues [43,49,50]. In addition, research has shown that terpenes and phenylpropanoids, commonly emitted by various fruits, play an important role in host-selection behavior [43,54], serving as chemical signals for insect recognition. Although mating behavior in tephritids varies by species [8], *A. fraterculus* and *C. capitata* are species that form leks—that is, aggregations of males formed exclusively for mating that intensify their attraction to females, resulting in greater chances of reproduction. For example, eugenol derivatives act as highly attractive kairomones for male tephritid [8] and are used as an indicator of mating sites. In fact, terpenes (myrcene) and terpenoids (linalool and geraniol) are precursors of the sex pheromone of *C. capitata*, which may explain their attractiveness to fruit flies [49,50]. Corroborating this, in a no-choice scenario, we showed that all tested compounds (α-pinene, β-pinene, ρ-cymene, β-caryophyllene, isoeugenol, γ-terpinene, coumarin, citronellal, geropine, and methyl cinnamate) did not reduce oviposition punctures in grape berries by *A. fraterculus* and *C. capitata*, when compared to the insecticide Delegate™ 250 WG. Although these terpenes and phenylpropanoids did not differ from grape berries that contained none of these compounds, we hypothesize that their efficacy can be enhanced when synergized with multiple phytoconstituents, thereby mimicking the odor profiles of plants, as already demonstrated in the “attract and kill” technique [8,58].

In addition to the toxicity of the tested compounds against *A. fraterculus* and *C. capitata*, it is also important to highlight their selectivity toward non-target organisms used in the management of these tephritids [4,32]. According to our results, all the compounds analyzed caused low mortality in *D. aerolatus* adults through topical application and ingestion bioassays, when compared to the synthetic insecticide Delegate™ 250 WG. Furthermore, β-pinene, γ-terpinene, and geropine did not differ from the distilled water treatment in the ingestion bioassay. This fact is of fundamental importance for the management of *A. fraterculus* and *C. capitata* in the field, given that *D. aerolatus* is the most promising native parasitoid for the biological control of tephritids in Brazil [4,59]. Furthermore, when considering the sublethal effect on *D. aerolatus*, none of the treatments affected parasitism. Previous studies have indicated the parasitic potential of *D. aerolatus* on larvae of *A. fraterculus* and *C. capitata* [4], demonstrating its potential in biological control programs for both species. These results suggest that tested phytoconstituents may have potential for integration with biological control strategies without significantly impairing parasitoid performance. This compatibility is particularly relevant because some insecticides used for tephritids management may exert lethal or sublethal effects on beneficial parasitoids, potentially compromising their contribution to integrated pest management programs [32].

## 5. Conclusions

The findings contribute to advancing our understanding of the insecticidal and behavioral potential of isolated terpenes and phenylpropanoids against economically important tephritids, highlighting differences in efficacy associated with chemical class, route of exposure, and target species. In particular, citronellal and isoeugenol stood out for their high toxicity, in some cases comparable to the commercial reference insecticide, while monoterpene hydrocarbons showed lower efficacy when evaluated in isolation, reinforcing the importance of synergistic interactions among such volatile phytochemicals. The higher toxicity observed via ingestion reinforces the applicability of these compounds in “attract and kill” management strategies, especially when combined in blends that mimic the natural chemical signals of host plants. Furthermore, the low toxicity and absence of relevant sublethal effects on the parasitism of *D. aerolatus* indicate a promising selectivity profile, compatible with integrated management programs for tephritids. Thus, the results obtained support the potential use of these compounds as alternative and environmentally safer tools for the control of *A. fraterculus* and *C. capitata*, encouraging future studies aimed at formulating synergistic mixtures, conducting field evaluations, and gaining a deeper understanding of their mechanisms of action.

## Figures and Tables

**Table 1:** Estimates of the LC_50_ and LC_90_ values (mg L^−1^) and confidence intervals of isolated compounds and a spinetoram-based synthetic insecticide (Delegate™ 250 WG™) to *Anastrepha fraterculus* and *Ceratitis capitata* adults in an ingestion bioassay (120 h of exposure).

Treatments	Slope ± SE	LC_50_ (CI 95%) ^a^	LC_90_ (CI 95%) ^b^	χ^2 c^	*df* ^d^
***A. fraterculus***					
β-pinene	2.45 ± 0.71	23.40 (18.11–26.07) ab *	105.48 (91.95–133.86) a	4.37	7
isoeugenol	3.11 ± 0.34	27.34 (23.77–33.45) b	96.12 (87.13–101.34) a	6.11	7
β-caryophyllene	2.90 ± 0.27	31.24 (29.73–35.11) b	99.13 (93.14–107.18) a	7.19	7
citronellal	3.14 ± 0.29	30.89 (28.10–34.11) b	87.13 (84.15–95.11) a	8.04	7
γ-terpinene	3.08 ± 0.11	37.13 (31.14–42.18) b	101.43 (96.17–111.24) a	9.16	7
geropine	3.12 ± 0.45	35.18 (30.17–40.16) b	114.53 (110.93–117.89) a	9.10	7
coumarin	3.14 ± 0.23	42.19 (38.19–49.10) b	123.34 (117.89–135.67) a	7.18	7
α-pinene	2.79 ± 0.45	33.44 (30.23–37.69) b	102.54 (98.13–119.10) a	9.03	7
methyl cinnamate	3.15 ± 0.67	39.10 (37.14–44.15) b	117.89 (112.32–130.14) a	9.45	7
ρ-cymene	3.12 ± 0.80	42.12 (38.19–46.17) b	109.80 (100.90–112.35) a	7.13	7
Delegate™ 250 WG	2.17 ± 0.93	17.11 (15.14–19.18) a	87.13 (83.12–92.118) a	6.17	7
***C. capitata***					
β-pinene	3.02 ± 0.34	39.10 (33.44–43.12) a	119.32 (114.12–132.10) b	8.74	7
isoeugenol	3.10 ± 0.75	29.11 (27.89–32.25) a	124.56 (111.32–132.23) b	9.12	7
β-caryophyllene	3.44 ± 0.89	33.34 (29.10–39.11) a	118.19 (114.32–122.43) b	8.03	7
citronellal	3.01 ± 0.92	30.13 (28.10–33.44) a	129.10 (122.35–135.67) b	7.19	7
γ-terpinene	2.78 ± 0.68	35.67 (32.14–40.13) a	131.45 (125.21–134.23) b	7.90	7
geropine	3.12 ± 0.24	33.44 (30.14–37.18) a	127.89 (122.19–134.12) b	8.04	7
coumarin	3.04 ± 0.34	32.14 (29.70–37.13) a	119.80 (117.89–123.48) b	9.34	7
α-pinene	2.99 ± 0.36	36.18 (32.17–39.10) a	123.47 (119.20–128.73) b	6.98	7
methyl cinnamate	3.12 ± 0.44	33.44 (29.13–38.12) a	129.35 (120.34–122.47) b	7.12	7
ρ-cymene	3.14 ± 0.23	30.19 (27.18–36.17) a	132.44 (128.19–135.62) b	8.10	7
Delegate™ 250 WG	2.99 ± 0.67	21.14 (19.10–33.45) a	74.51 (70.11–81.12) a	7.90	7

Concentrations (mg L^−1^) required to kill 50 (CL_50_) ^a^ or 90% (CL_90_) ^b^ of the adults of *A. fraterculus* and *C. capitata*, respectively (CI: confidence interval at 95% error probability); ^c^ χ^2^: Pearson’s chi-square value; ^d^ df: degrees of freedom. * Significance of differences among slopes determined by likelihood ratio test of equality followed by pairwise comparisons using non-overlapping fiducial limits.

**Table 2:** Estimates of the LC_50_ and LC_90_ values (mg L^−1^) and confidence intervals calculated 120 h after the topical application bioassays with pure compounds and the spinetoram-based synthetic insecticide (Delegate 250™ WG) against adults of *Anastrepha fraterculus* and *Ceratitis capitata*.

Treatments	Slope ± SE	LC_50_ (CI 95%) ^a^	LC_90_ (CI 95%) ^b^	χ^2 c^	*df* ^d^
***A. fraterculus***					
β-pinene	3.45 ± 0.71	33.40 (31.46–34.07) b *	115.48 (110.95–123.86) b	8.37	7
isoeugenol	2.77 ± 0.23	32.11 (29.10–34.16) b	121.34 (117.89–124.35) b	7.11	7
β-caryophyllene	3.11 ± 0.44	35.16 (31.11–37.19) b	117.89 (115.13–121.34) b	8.17	7
citronellal	4.02 ± 0.32	32.10 (29.10–33.44) b	120.23 (118.90–127.80) b	9.10	7
γ-terpinene	3.78 ± 0.45	37.18 (34.11–40.19) b	119.10 (115.67–123.47) b	7.18	7
geropine	3.66 ± 0.77	35.17 (33.16–38.19) b	123.56 (120.11–130.23) b	6.56	7
coumarin	2.90 ± 0.63	29.10 (27.19–33.12) b	127.89 (122.35–131.24) b	8.19	7
α-pinene	2.87 ± 0.43	35.16 (30.12–39.10) b	122.36 (119.03–127.87) b	7.87	7
methyl cinnamate	2.97 ± 0.54	33.14 (31.44–37.19) b	118.90 (116.45–125.67) b	7.18	7
ρ-cymene	3.11 ± 0.67	34.17 (30.12–38.19) b	124.57 (120.34–131.25) b	8.19	7
Delegate™ 250 WG	2.98 ± 0.89	21.44 (19.74–24.15) a	90.11 (87.11–93.12) a	7.10	7
***C. capitata***					
β-pinene	2.90 ± 0.32	30.18 (29.17–34.12) b	125.65 (120.11–131.23) b	6.13	7
isoeugenol	3.17 ± 0.87	31.44 (30.15–33.15) b	119.13 (114.23–125.67) b	8.10	7
β-caryophyllene	3.18 ± 0.99	29.76 (28.19–33.12) b	122.56 (119.34–127.43) b	9.03	7
citronellal	3.45 ± 0.56	34.15 (32.19–36.11) b	131.45 (128.77–132.12) b	7.19	7
γ-terpinene	2.87 ± 0.99	31.45 (29.10–32.19) b	124.12 (118.90–128.34) b	8.10	7
geropine	3.14 ± 0.12	33.10 (31.15–36.78) b	132.18 (129.11–134.56) b	9.13	7
coumarin	2.96 ± 0.35	31.76 (29.10–34.12) b	144.23 (140.13–147.11) b	8.19	7
α-pinene	3.14 ± 0.67	30.15 (28.10–34.12) b	129.13 (126.18–134.67) b	7.14	7
methyl cinnamate	2.96 ± 0.71	29.10 (27.18–32.18) b	134.56 (129.11–141.28) b	8.10	7
ρ-cymene	3.12 ± 0.44	33.11 (29.30–37.18) b	129.10 (127.89–136.78) b	9.03	7
Delegate™ 250 WG	3.10 ± 0.53	23.17 (20.11–26.18) a	87.11 (85.68–90.13) a	7.18	7

^a^ LC_50_ and ^b^ LC_90_: Insecticide concentrations (mg L^−1^) required to kill 50 or 90% of the adults of *A. fraterculus* and *C. capitata*, respectively (CI: confidence interval at 95% error probability); ^c^ χ^2^: Pearson’s chi-square value; ^d^ df: degrees of freedom. * Significance of differences among slopes determined by likelihood ratio test of equality followed by pairwise comparisons using non-overlapping fiducial limits.

**Table 3:** Number of punctures (means ± SE) caused by *Anastrepha fraterculus* and *Ceratitis capitata* larvae on grape berries treated by immersion with various isolated compounds and a spinetoram-based synthetic insecticide (Delegate™ 250 WG).

Treatments	Number of Punctures by *A. fraterculus*	Number of Punctures by *C. capitata*
β-pinene	1.32 ± 0.17 a	0.75 ± 0.12 a
isoeugenol	1.44 ± 0.22 a	0.82 ± 0.23 a
β-caryophyllene	1.40 ± 0.34 a	0.76 ± 0.17 a
citronellal	1.35 ± 0.23 a	0.89 ± 0.12 a
γ-terpinene	1.45 ± 0.18 a	0.82 ± 0.08 a
geropine	1.30 ± 0.14 a	0.77 ± 0.11 a
coumarin	1.46 ± 0.23 a	0.88 ± 0.13 a
α-pinene	1.44 ± 0.32 a	0.81 ± 0.21 a
methyl cinnamate	1.55 ± 0.17 a	0.82 ± 0.17 a
ρ-cymene	1.39 ± 0.18 a	0.79 ± 0.04 a
Delegate™ 250 WG	0.00 ± 0.00 b	0.00 ± 0.00 b
Distilled water	1.89 ± 0.13 a	1.10 ± 0.09 a
F	22.45	17.56
*df*	11, 300	11, 300
*p* values	<0.0001	<0.0001

Means followed by different letters in the columns indicate significant differences between treatments (GLM with a quasi-Poisson distribution followed by Tukey’s post hoc test, *p* < 0.05).

**Table 4:** Mortality (%) (mean ± standard error) and parasitism (P, %) of *Doryctobracon areolatus* 120 h after exposure to treatments in the laboratory.

Treatments	Mortality (%)		P (%) ^a^
	Ingestion Bioassay ^a^	Topical Application Bioassay ^a^	
β-pinene	4.11 ± 0.87 ab	14.23 ± 0.78 b	44.07 ± 6.19 a
isoeugenol	13.15 ± 0.35 b	12.35 ±1.14 b	62.13 ± 7.10 a
β-caryophyllene	11.76 ± 0.59 b	11.09 ± 0.96 b	49.11 ± 3.98 a
citronellal	10.98 ± 0.87 b	14.24 ± 1.13 b	54.12 ± 8.10 a
γ-terpinene	6.12 ± 0.76 ab	13.82 ± 0.87 b	56.17 ± 5.67 a
geropine	7.18 ± 0.93 ab	12.76 ± 1.11 b	49.04 ± 6.03 a
coumarin	10.92 ± 1.14 b	11.65 ± 0.89 b	43.21 ± 4.10 a
α-pinene	9.11 ± 0.83 b	10.44 ± 0.97 b	46.17 ± 5.16 a
methyl cinnamate	17.12 ± 0.67 b	11.80 ± 1.14 b	57.82 ± 7.02 a
ρ-cymene	14.15 ± 1.16 b	13.90 ± 1.03 b	48.02 ± 9.16 a
Delegate™ 250 WG	38.10 ± 0.95 c	31.90 ± 0.99 c	56.78 ± 5.78 a
Distilled water	1.12 ± 0.44 a	0.90 ± 0.03 a	51.32 ± 4.89 a
F	9.11	10.12	17.90
*dfl*	11, 240	11, 240	11, 240
*p* values	<0.0001	<0.0001	<0.0001

Means followed by different letters in the columns indicate significant differences between treatments (GLM with a quasi-Poisson distribution followed by Tukey’s post hoc test, *p* < 0.05).

## Data Availability

The original data are included in the paper. Further inquiries can be directed to the corresponding author.
